# Papermills as another challenge to research integrity and trust in science

**DOI:** 10.3389/fmed.2025.1557024

**Published:** 2025-02-28

**Authors:** Arch G. Mainous

**Affiliations:** ^1^Department of Community Health and Family Medicine, University of Florida, Gainesville, FL, United States; ^2^Department of Health Services Research Management, and Policy, University of Florida, Gainesville, FL, United States

**Keywords:** trust, research integrity, papermill, artificial intelligence, editors

## Introduction

Evidence has continued to accumulate showing the decreasing trust by the general public in government health agencies (1, 2). The general public in the United States and other countries in Europe and Asia has exhibited declining trust in government health agencies and their health recommendations (2–5). The trust in health agencies is built on an assumption that the science that underpins their health recommendations is solid and the evidence is trustworthy.

Medical journals need to reinforce the research integrity of the studies that they publish to ensure the confidence in the evidence from everyone who reads and acts upon those studies. Research integrity policies followed by medical journals ensure that the research that is published is conducted ethically and upholds the highest standards of scientific credibility and trustworthiness within the medical field.

In addition to guarding against scientific fraud and not publishing poor quality research, a new threat that threatens the credibility and trustworthiness of published medical studies has arisen that is an insidious theat. Papermills are the process by which manufactured manuscripts are submitted to a journal for a fee on behalf of researchers with the purpose of providing an easy publication for them, or to offer authorship for sale (6). Buying authorship on a paper can help with career advancement. The old adage of “publish or perish” still holds critical importance for many academics around the world and thus the papermills can exploit that fear. These papers are not necessarily filled with fraudulent data but many of these papers have low scientific value. The goal is not to add to our knowledge base or push the field forward but rather to get a peer reviewed publication, the currency of advancement and promotion in academics and particularly in many low and middle-income countries.

## The advantages of large databases and the potential of exploitation by papermills

Although not mentioned in some overviews of papermills is the use of publicly available data for papers (6). Rather than using faked data or manipulated images in something like a cell biology paper, using publicly available data like the large databases available from the United States' National Center for Health Statistics can provide instant credibility for a paper. Databases like the National Health and Nutrition Examination Survey (NHANES) are highly respected and provide hundreds of variables for use in making population-based estimates of the US population. Many studies have used these databases because of their size (hundreds of variables; thousands of people), diversity of measures (questionnaires; laboratory tests; physical examinations), and importantly the characteristic that the data is freely available and can be instantly downloaded by anyone in any country.

These databases have been the basis for many important studies and prevalence assessments (7–10). However, because of the large number of variables that are available it seems that enterprising groups can build a matrix of variables and simply correlate the variables looking for statistically significant relationships. What is missing in these analyses is a hypothesis with clear outcomes. Further, the hypothesis would need to consider contextual factors that are the underpinnings of the NHANES data set or other US data set and the need for cultural adaptation in other global cultural domains. Thus, the question of does the NHANES data set apply to the cultural context of the country of origin of the manuscript, and therefore does the hypothesis answer a critical question are questions that need to be used by reviewers and editors in evaluating manuscripts. Unfortunately, these scientifically dubious but statistically significant relationships can be used as the basis for manuscripts to be sold by papermills. By having statistically significant relationships the likelihood of publication is enhanced. It is important to remember that if enough comparisons are computed some will be statistically significant even if it is purely by chance.

This creates a deluge of papers that can be sent to journals overwhelming editors and reviewers. Unfortunately, many unsophisticated peer reviewers will focus on the statistically significant results and not critically consider the scientifically dubious mechanism or pathway proposed between the two variables that was created in a *post hoc* manner to justify the study itself.

Mendelian randomization is another strategy to leverage publicly available data and use it to create a vast number of papers, many with little scientific value (11). In particular, many studies use two-sample Mendelian randomization (2SMR) designs. The accessibility of this data has yielded an avalanche of papers using 2SMR sent to medical journals which have little scientific rigor and which are scientifically dubious. A variety of these 2SMR studies are being produced in papermill organizations that are then sold to individuals needing authorship on peer-reviewed papers (11). Again, it is important for reviewers and editors to consider the scientific value of the paper presented to them.

## Strategies to improve research integrity by *Frontiers in Medicine*

*Frontiers in Medicine* and *Frontiers* in general, has tried to combat papermills and increase confidence in the results published in scientific manuscripts via several strategies. First, the proprietary artificial intelligence system used by *Frontiers in Medicine*, AIRA can give cues on papermills and patterns that humans can't easily see. Further, papermills tend to send out papers on the same topic, including the exact same paper, to many different journals simultaneously. Individual editors and reviewers don't have the ability to see multiple papers simultaneously but AIRA can. Second, even though papermills will have multiple versions of the same paper with different author names and different author institutions AIRA can detect this and even see how the papers can be part of a network. Third, AIRA helps with 60 different checks prior to peer review. These include language evaluation, image integrity verification, frontiers manuscript matches, ethics statement verification, commercial keyword detection, and potential controversial themes. In 2024, more than half of all article rejections were performed by the research integrity team, thus ensuring quality controls are upheld and alleviating the workload for the editorial boards.

An important consideration is that AI only knows as much as it is taught. Thus, the human factor which picks up on new themes and new methodologies needs to be nimble and alert for changes in strategy by the papermills. It is critical to remember that papermills are businesses and will be constantly looking for ways to sell their products. Until AIRA has a chance to learn the new methodologies, it will not be useful in identifying them so the research integrity teams need to constantly be on the lookout for new strategies by papermills.

## Analysis of the NHANES as an example of a data source for potential papermill papers

As was mentioned earlier, the NHANES is a large, highly credible database of a nationwide probability sample of the US population conducted by the National Center for Health Statistics. The first NHANES was conducted in 1971. A Pubmed search on January 1, 2025 using the search term “NHANES” yielded >74,900 different citations. Moreover, there were more than 7,400 citations in 2024. Interestingly, considering that the NHANES is designed to make population-based estimates of the US population, investigators from other countries are aggressively using this data. A PubMed search for 2024 using “NHANES” and “USA” yielded just over 1,000 papers. The use by investigators from other countries varies significantly. Authors from France were listed on fewer than 125 papers, and authors from the United Kingdom were listed on fewer than 190 papers. On the other hand, a search with “NHANES” and “China” yields >3,800 papers. Thus, even though the NHANES is designed for estimates of the US population, investigators from China account for half of the NHANES publications in 2024 and 3 times as many NHANES articles as investigators from the US.

**Figure 1:**
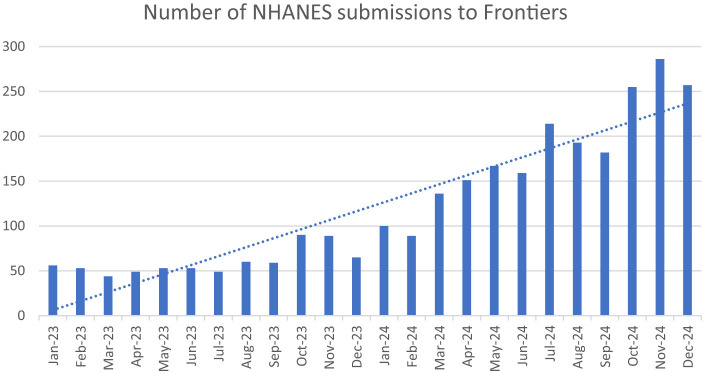
Number of submissions using NHANES data to *Frontiers* in 2023 and 2024.

The experience with an increase in NHANES submissions at *Frontiers* journals (not just *Frontiers in Medicine)*, shows a significant increase in submissions in the past year (Figure 1). There were 56 NHANES submissions to *Frontiers* in January 2023 and 286 in November 2024. A high proportion of NHANES submissions go on to be rejected, mostly by the editorial office for integrity concerns. Of the NHANES submissions to *Frontiers in Medicine* in 2024, 60 have been rejected. Fifty three of these rejections were performed by the editorial office, of these rejections, 46 were for general integrity concerns, including papermill suspicion, and of these 19 were rejected solely on evidence of being products of papermills.

## Where do we go from here?

*Frontiers in Medicine* has editorially rejected a variety of papers that come from papermills. These include basic science studies and those with fraudulent data. Further, studies that use publicly accessed data analyzed in Mendelian randomization studies, and large databases like the NHANES have also been rejected as products of papermills. The variables or signals that increase suspicion of a paper coming from papermills is not shared publicly. The reason for this control of information on specific papermill signals, is that it is likely that papermill companies would use this knowledge to circumvent the checks at the journal.

Papermills may not be of particular relevance to many investigators in Western industrialized countries. Even if that it is so, papermills are a problem for scientific journals and the general knowledge base that drives medical decisions. Observations that are made on US derived data sets by authors who are not within the US healthcare or policy milieu, and possibly not familiar with the nuances of US healthcare, may generate great papers that lack the contextual relevance for policy-or healthcare-relevant conclusions.

As valuable as artificial intelligence can be in this process, it is important for scientists who are experts in the area to judge these papers on their scientific plausibility and basis for the proposed mechanism or pathway. The papermills can overwhelm editors and reviewers with the sheer volume of papers. It is easy to be swayed by seeing a statistically significant relationship and not critically assess whether it makes any sense. With so many different variables and different laboratory tests and diseases available in these big databases for analysis a matrix of variables will find some “new” relationships even if they are non-sensical under critical assessment. The papermills are counting on editors and reviewers focusing on the *p* value and not looking at the actual underlying science of the study. It is incumbent on editors, reviewers and AI to identify these papers and reject them to help and ensure research integrity and corresponding trust in science.
